# Integrated analysis identifies key genes underlying the bidirectional association between depression and renal failure

**DOI:** 10.1038/s41598-025-04707-9

**Published:** 2025-07-01

**Authors:** Zhengqi Qiu, Yu Nie, Xu Lin, Jun Du, Juntao Kan, Emma Yun Zhi Huang

**Affiliations:** 1 The Institute of Mental Psychology, School of Health Management, Guangzhou Medical University, Guangzhou, 510370 China; 2 School of Public Health, Sun Yat-sen University, No.74, 2nd Zhongshan Road, 510080 Guangzhou, China; 3 Amway R&D Center, Nutrilite Health Institute, Building 6, No.720, Cailun Road, Pudong New Area, Shanghai, 201203 China

**Keywords:** Bioinformatics, Depression, End-stage renal disease, Immune system, UK biobank, WGCNA, Genome informatics, Predictive medicine, Psychology, Kidney diseases

## Abstract

Depression is a common psychiatric comorbidity in individuals with end-stage renal disease (ESRD). However, the underlying biological mechanisms and the precise relationship between depression and renal failure remain unclear. While interventions such as cognitive behavioral therapy and exercise have been shown to alleviate symptoms, the interplay between these conditions and their molecular pathways is poorly understood. An integrated analysis was conducted combining bioinformatics approaches and data from the UK Biobank (UKB) cohort. The UKB study revealed a significant association between renal failure and depression. Gene expression data from the Gene Expression Omnibus (GEO) database were analyzed to identify key co-expression modules using Weighted Gene Co-expression Network Analysis (WGCNA). Protein-protein interaction (PPI) networks were constructed using the STRING database, and immune cell infiltration was assessed with the CIBERSORT tool. UKB data confirmed a robust association between renal failure and depression. Bioinformatics analyses highlighted significant enrichment in pathways related to the acute inflammatory response, specific granule lumen, and immune receptor activity. PPI network analysis identified 23 hub genes, including *CYP4F2*, *KCNA3*, *KISS1R*,* LILRA5*, and *ZC3H12D*, as key players in the shared pathophysiology of ESRD and depression. Validation studies further emphasized the roles of *LILRA5*, *CYP4F2*, and *KISS1R* in these mechanisms. This study reveals novel insights into the molecular and immune interactions underlying the comorbidity of renal failure and depression. By combining cohort and bioinformatics analyses, we identify potential therapeutic targets and pathways that may inform innovative treatment strategies.

## Introduction

Depression is widely recognized as one of the most burdensome mental health disorders worldwide, significantly impairing daily functioning over prolonged periods^1^. In the United States alone, depression—including major depressive disorder (MDD), bipolar disorder, and dysthymia—was estimated to cost $83.1 billion in 2000^2^. According to the World Health Organization, 322 million people globally suffer from MDD, reflecting an 18.4% increase between 2005 and 2015^3^. Moreover, depression among adolescents and older adults further elevates medical burdens, a challenge expected to worsen during the COVID-19 pandemic^4,5^. Concurrently, the rising prevalence of end-stage renal disease (ESRD) has led to around 100,000 Americans awaiting kidney transplants. In 2016, approximately 13,000 patients received a deceased donor organ; half had been on dialysis for over five years^6^. Patients with chronic kidney disease (CKD) who also experience depression face heightened risks of negative clinical outcomes, including accelerated declines in estimated glomerular filtration rate (eGFR), earlier initiation of dialysis, and reduced life expectancy^7^. Consequently, early screening and treatment for depression are often recommended for individuals with CKD^8,9^. Indeed, evidence suggests that depression is one of the most common psychiatric conditions among people with ESRD. Its prevalence in the dialysis population ranges from 22.8% (based on interview-based diagnoses) to 39.3% (according to self- or clinician-administered rating scales), likely owing to overlapping symptoms of uremia and depression^10^.

It is hypothesized that several pathophysiological processes may link depression and renal failure. First, chronic renal failure is characterized by persistent systemic inflammation, which may induce a uremic phenotype encompassing depression, cardiovascular disease, and protein-energy depletion^11^. Prolonged systemic inflammation also appears to increase the likelihood of depression in patients with renal failure, potentially explaining the high depression rates in this population^12^. Additionally, the accumulation of uremic toxins may contribute to dialysis encephalopathy, particularly if an impaired blood-brain barrier leads to mild confusion, sleep disturbances, and other neurological dysfunctions^13^. Further, elevated triglycerides in chronic renal failure could disrupt the brain’s regulation of cortisol levels, and high triglyceride levels have been linked to bipolar disorder (manic depression)^14,15^. Although the precise pathophysiological mechanisms remain unclear, certain genetic and molecular biological processes may underlie both major depression and renal failure.

Existing studies have shown that approximately 27% of patients with CKD and end-stage kidney disease (ESKD) experience comorbid depression, which is significantly associated with reduced quality of life and increased mortality^16,17^. In older adults, depression often coexists with cognitive impairment, thereby compounding disease burden^18,19^. Some evidence suggests that inflammation may play a role in the pathophysiology of depression in CKD/ESKD; however, the underlying mechanisms require further investigation^20^. Notably, depression can accelerate type 2 diabetes–related kidney damage and increase the risk of progressing to end-stage renal disease (ESRD) and pre-ESRD mortality^21^. In addition, hematological indices such as the neutrophil-to-lymphocyte ratio and the platelet-to-lymphocyte ratio (PLR) have been proposed as convenient biomarkers for aiding depression diagnosis^22^. In specific populations—such as Australian Aboriginal and Torres Strait Islander peoples—it is crucial to consider multiple psychosocial stressors that may negatively affect emotional well-being^23^. Consequently, future large-scale prospective studies are warranted to clarify the links between depression and kidney disease and to develop targeted interventions for managing multiple comorbidities, ultimately aiming to improve patient outcomes and quality of life.

This study investigated molecular mechanisms underlying kidney failure–related depression by identifying key genes commonly altered in both conditions. Differentially expressed genes were analyzed through co-expression analysis, pathway enrichment analysis, and immune cell infiltration assessment. Additionally, analyses of large prospective cohorts from the UK Biobank (UKB) demonstrated a significant bidirectional association between depression and kidney failure, emphasizing the clinical importance of investigating this comorbidity. Potential diagnostic biomarkers and immunotherapeutic targets for this comorbidity were also explored.

## Materials and methods

### Data sources

In this study, gene expression profiles for both depression and renal failure were sourced from the publicly available Gene Expression Omnibus (GEO) database (www.ncbi.nlm.nih.gov/geo). For depression, the GSE98793 dataset was utilized, which includes 192 whole blood samples: 64 from healthy controls, 64 from patients with simple major depressive disorder (MDD), and 64 from patients with MDD accompanied by anxiety disorders. Given that major depression (MDD) was the primary focus of this study, only samples from patients with simple MDD were retained for analysis.

For renal failure, we used the GSE37171 dataset, comprising 115 peripheral blood samples: 40 from healthy controls and 75 from patients diagnosed with chronic renal failure. Both datasets were generated using the GPL570 platform (HG-U133_Plus_2; Affymetrix Human Genome U133 Plus 2.0 Array), ensuring consistent and comparable gene expression data across both cohorts. Additionally, the clinical data obtained from the UKB were integrated to explore the bidirectional association between depression and renal failure in a large, prospective cohort. The UK Biobank provides comprehensive data on participants’ health, lifestyle, and sociodemographic factors, including diagnoses for both depression (using ICD-10 codes F31.3, F31.4, F31.5, F32, F33) and renal failure (using ICD-10 codes N17-N19, E85.3, N16.5, etc.), which were leveraged to assess clinical outcomes. This combination of bioinformatics data and cohort-based clinical information forms the basis for understanding the molecular and clinical intersections between depression and renal failure.

### Data preparation

R software (v4.2.2) and associated Bioconductor packages were employed for data preparation. Raw data were processed to generate expression matrices, followed by probe-to-gene symbol matching. Preprocessing and normalization were carried out with the Affy package, using interpolation to handle missing values^24^. For the UKB data, a structured data extraction approach was followed. Participants with depression (ICD-10 codes: F31.3, F31.4, F31.5, F32, F33) and renal failure (ICD-10 codes: N17-N19, E85.3, N16.5, etc.) were identified from the cohort. To account for potential confounders, variables such as age, sex, ethnicity, smoking status, alcohol consumption, BMI, and comorbid conditions were included in the statistical models. We applied the standardization procedure to numerical variables, ensuring comparability across participant subgroups. For clinical event analysis, the incidence of depression and renal failure was assessed using a Cox proportional hazards model. The UKB data were further processed using the UKB data analysis tools, with quality control measures implemented to ensure the accuracy and consistency of results, as outlined in previously published studies (Supplementary Table 1).

### Co-expression network construction

Weighted Gene Co-expression Network Analysis (WGCNA) identified gene modules through hierarchical clustering^25^. Gene co-expression networks comparing depression versus normal and renal failure versus normal groups were constructed using the WGCNA package (v1.73) in R.

Differential gene expression analysis was performed using the limma package (v3.60.6) in R^26^. Genes with minimal expression variability were excluded by setting the standard deviation (SD) threshold to 0.5. The “pickSoftThreshold” function was then applied to identify the soft-power value based on scale-free topology. The adjacency matrix was computed as $\:{{\upalpha\:}}_{\text{m}\text{n}}={\left|\text{c}\text{o}\text{r}\left({x}_{\text{m}},{x}_{\text{n}}\right)\right|}^{{\upbeta\:}}$($\:{{\upalpha\:}}_{\text{m}\text{n}}$: adjacency between gene m and gene n, $\:\text{c}\text{o}\text{r}$: Pearson’s correlation, and β: soft-power threshold), and transformed into a topological overlap measure (TOM) matrix for connectivity estimation.

### Functional enrichment analysis of common genes

To analyze the overlap of genes between the depression and renal failure groups, a Venn diagram was generated using Venn Diagram (v1.7.3). Functional enrichment analyses, including Gene Ontology (GO) and Kyoto Encyclopedia of Genes and Genomes (KEGG) pathway enrichment, were conducted on genes of interest. GO and KEGG analyses were performed using DAVID (https://david-d.ncifcrf.gov/), with significance set at *P* < 0.05. Additionally, Gene Set Enrichment Analysis (GSEA) was conducted using the clusterProfiler package (v4.4.4), referencing the c2.cp.all.v2022.1.Hs.symbols.gmt gene set. Parameters included num = 100, weighted scoring, and thresholds of nominal *P* < 0.01, FDR < 0.01, and |NES| (Normalized Enrichment Score) > 1 for significance.

### Mining crucial genes and identifying hub genes in functional modules

The STRING database (v11.5, http://string-db.org/) was used to identify common genes associated with depression and renal failure. Protein-protein interaction (PPI) networks were constructed and visualized using Cytoscape (v3.9.1). The CytoHubba plugin in Cytoscape was used to apply the Maximum Clique Centrality (MCC) algorithm to identify hub genes, which were defined as those with the highest interaction scores and considered central to disease co-morbidity^27^. The GeneCards database (http://www.genecards.org/) was further utilized to explore interactions of hub genes with related genes, proteins, drugs, and diseases^28^.

### Identification and correlation of disease immune infiltrate cells

The immune microenvironment includes immune cells, fibroblasts, cytokines, and chemokines, which influence disease progression and treatment response. Using CIBERSORT algorithm estimated the abundance of 22 immune cell types. Immune cell compositions were visualized with violin plots, and differences were assessed using the Wilcoxon rank-sum test (*P* < 0.05)^29^.

### Drug-target analysis

Potential drug-gene interactions involving key genes related to depression and renal failure were analyzed using the Enrichr platform (https://maayanlab.cloud/Enrichr/), specifically the Drug Signature Database (DSigDB) library, which compiles known associations between drugs and gene expression profiles^30^.

## Results

### Bidirectional associations between depression and incident renal failure

The results from the UK Biobank data analysis revealed significant bidirectional associations between depression and renal failure (Table 1). Participants with depression at baseline exhibited a higher incidence of renal failure compared to those without depression. Specifically, individuals with depression had an incidence rate of 8.39 per 1000 person-years, significantly higher than the 5.48 per 1000 person-years observed in those without depression. In Model 1, which was adjusted for age and sex, the hazard ratio (HR) for incident renal failure among participants with depression was 1.99 (95% CI: 1.92–2.07). After adjusting for potential confounders such as age, sex, and socio-economic factors (Model 2), the HR decreased to 1.42 (95% CI: 1.37–1.47), and in the fully adjusted model (Model 3), it further reduced to 1.40 (95% CI: 1.35–1.45), suggesting a persistent but attenuated association between depression and the risk of renal failure.

**Table 1:** Bidirectional associations of depression and renal failure among participants from the UK Biobank.

Outcome	Exposure	*N*	Cases	Incident rate (per 1000 person-years)	HR (95% CI)		
					Model 1^a^	Model 2^b^	Model 3^c^
Incident renal failure	Depression						
Incident depression	No	469,436	31,501	5.48	1 (reference)	1 (reference)	1 (reference)
	Yes	30,989	3093	8.39	1.99 (1.92–2.07)	1.42 (1.37–1.47)	1.40 (1.35–1.45)
	Renal failure						
	No	495,917	25,240	4.17	1 (reference)	1 (reference)	1 (reference)
	Yes	1,869	170	8.99	2.38 (2.05–2.77)	1.77 (1.52–2.07)	1.60 (1.37–1.87)

^a^Model 1 adjusted for age and sex.

^b^Model 2 adjusted for age, sex, Townsend deprivation index, ethnicity, assessment center, education level, BMI, sleep duration, smoking status, alcohol intake frequency, healthy diet score, grip strength, HbA1C, eGFR, and urate.

^c^Model 3 adjusted for age, sex, Townsend deprivation index, ethnicity, assessment center, education level, BMI, sleep duration, smoking status, alcohol intake frequency, healthy diet score, grip strength, HbA1C, eGFR, urate, SBP, use of diabetes medication, use of cholesterol-lowering medication, use of antihypertensive medication, history of coronary heart disease, and history of cancers.

BMI, body mass index; CI, confidence interval; eGFR, estimated glomerular filtration rate; SBP, systolic blood pressure.

On the other hand, the incidence of depression was also significantly higher in individuals who developed renal failure. Participants with renal failure exhibited an incidence rate of 8.99 per 1000 person-years of depression, compared to 4.17 per 1000 person-years in those without renal failure. In the unadjusted model, the HR for incident depression in individuals with renal failure was 2.38 (95% CI: 2.05–2.77). After adjusting for confounding factors (Model 2), the HR decreased to 1.77 (95% CI: 1.52–2.07), and in the fully adjusted model (Model 3), it further reduced to 1.60 (95% CI: 1.37–1.87).

### Construction of gene co-expression modules

Genes with a standard deviation ≥ 0.5 were included in the WGCNA analysis. A cluster analysis was conducted with two data sets without detecting any outliers: the disease group (depression and renal failure) and the control group. The scale independence and mean connectivity were shown in Supplementary Fig. 1A-B. Clustering dendrogram of genes with assigned module colors were shown in Supplementary Fig. 1C-D.

**Fig. 1:**
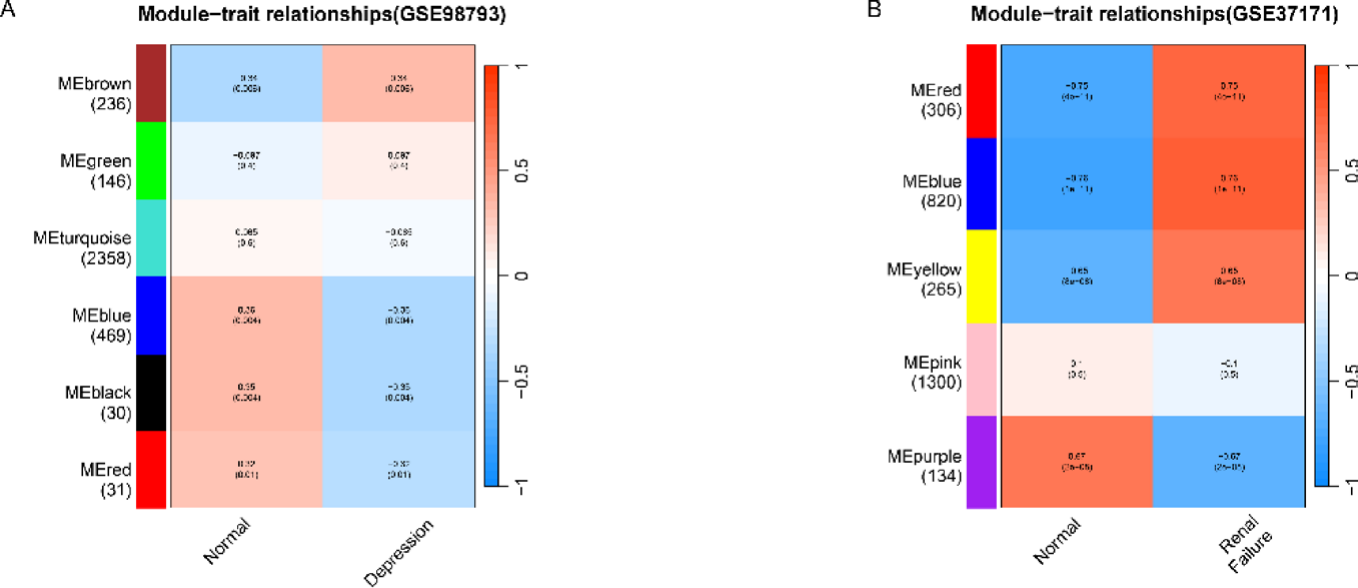
Identify the key modules related to depression and renal failure. (**A**, **B**) Heatmaps showing the correlation between the module eigengenes (MEs) and clinical traits of depression and renal failure. Each row represents an ME, and each column represents a clinical trait. Each cell displays the correlation coefficient and its corresponding P-value, illustrating the strength and statistical significance of the relationship between the module and clinical traits. The numbers in each row indicate the number of genes contained within each module.

In the WGCNA analysis of the depression dataset (GSE98793), the optimal soft-thresholding power was determined as 2, where R² reached approximately 0.9, achieving a good balance between scale-free topology and network complexity. For the renal failure dataset (GSE37171), although the estimated power value was relatively high and less biologically appropriate, we selected power = 2 for both datasets to ensure consistency and comparability across network construction. The genes in GSE98793 and GSE37171 were clustered into various modules using hierarchical clustering analysis and dynamic branch cut methods for gene dendrograms (Fig. 1A and B). The specific module partitioning details are provided in Supplementary File 1–2, while the analysis code can be found in Supplementary File 3.

### Calculation of module-trait correlations

Gene significance (GS) was defined as the absolute value of the correlation between individual gene expression profiles and the clinical trait. Module membership (MM) was calculated as the correlation between each gene’s expression and the corresponding module eigengene (ME), representing how well a gene fits within a module. Genes with high GS and MM values were considered intramodular hub genes, indicating both biological relevance to the phenotype and centrality within the co-expression module.

**Fig. 2:**
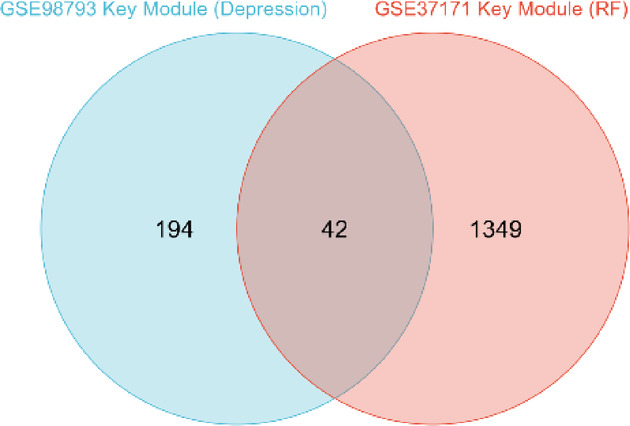
Venn diagram of the 42 common genes between depression and renal failure. An analysis of the key modules associated with depression and renal failure has identified common genes.

In our analysis, the brown module was specifically associated with depression, as illustrated in Fig. 1A, whereas renal failure was correlated with the red, blue, and yellow modules, as demonstrated in Fig. 1B. Subsequently, by intersecting the key modules of the two diseases, as illustrated in Fig. 2, we identified 43 genes that were co-expressed in depression and renal failure (Supplementary Table 2).

### Functional enrichment analysis of common genes

**Fig. 3:**
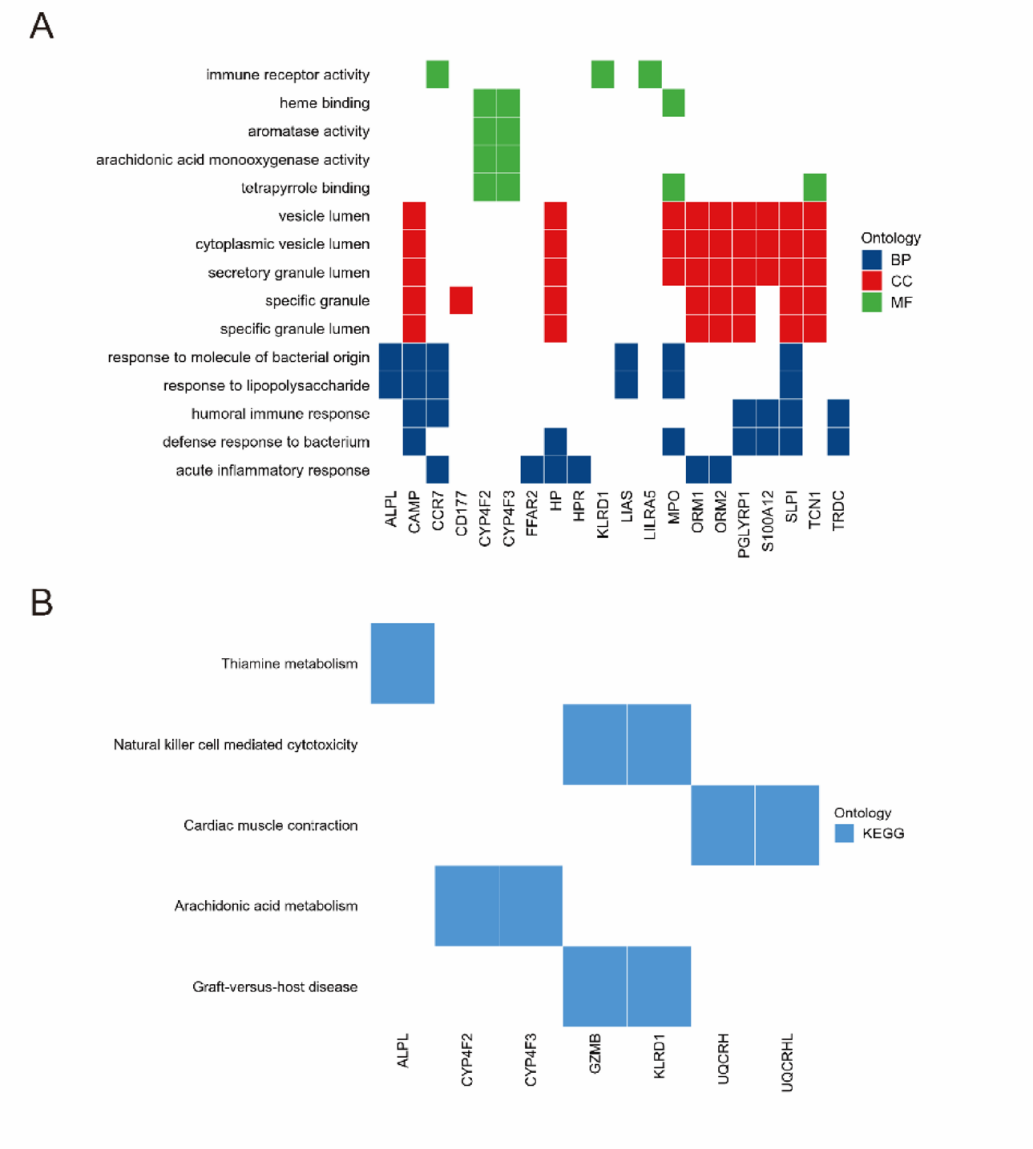
GO and KEGG analyses of common genes related to depression and renal failure in selected modules. (**A**) GO enrichment analysis of genes associated with biological processes (BP), cellular components (CC), and molecular functions (MF). (**B**) Heatmap illustrating the enrichment of KEGG pathways in the relevant molecules, highlighting the key biological pathways shared between depression and renal failure.

Our comprehensive GO and KEGG pathway enrichment analysis, as illustrated in Supplementary Tables 3 and Fig. 3, has effectively illuminated the complex molecular biological mechanisms shared by genes common to both depression and renal failure, providing insights into potential comorbidity mechanisms. The GO analysis categorized into Biological Processes (BP), Cellular Components (CC), and Molecular Functions (MF) has revealed significant findings.

In BP, the processes such as acute inflammatory response, response to lipopolysaccharide, and response to molecule of bacterial origin were considerably enriched, highlighting the role of immune and inflammatory responses in the pathology of both conditions. In CC, Top enrichments were observed in specific granule lumen, specific granule, and secretory granule lumen, suggesting a crucial role for these cellular structures in disease mechanisms. In MF, Enrichment in functions like immune receptor activity, tetrapyrrole binding, and serine-type endopeptidase activity underscores the importance of these molecular functions in mediating the biological processes involved in both diseases.

The KEGG pathway analysis further complements findings. Pathways such as Graft-versus-host disease, Natural killer cell mediated cytotoxicity, and Thiamine metabolism were among the top enriched pathways. These pathways point towards significant immune system involvement and metabolic dysregulation in the comorbidity of depression and renal failure.

### Identification of hub genes in depression and renal failure

**Fig. 4:**
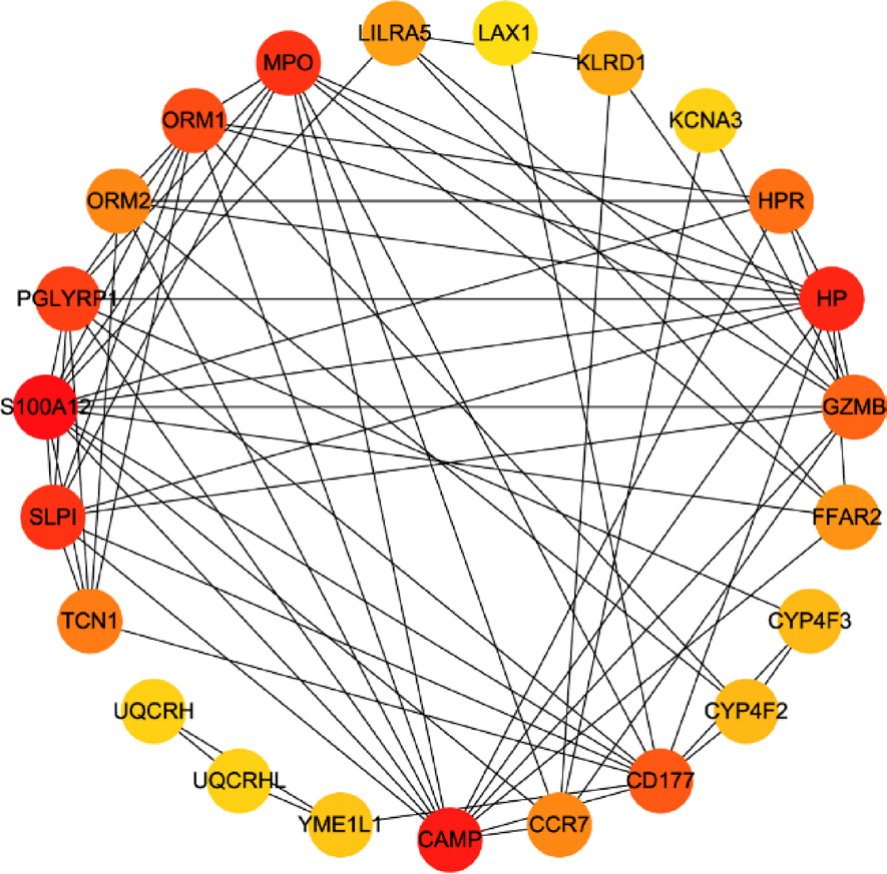
Protein-protein interaction network analysis of key module genes. Protein-protein interaction (PPI) networks of common genes in depression and renal failure were developed based on the STRING database. The 23 genes with the highest connectivity of MCC were visualized with Cytoscape software. Node color is directly proportional to the MCC of gene connectivity.

The PPI network was constructed based on experimentally validated interactions between depression and renal failure common genes with a combined score greater than 0.15 in the STRING database. The PPI network was visualized using the Cytoscape software (Fig. 4). A PPI network consists of 23 nodes and 74 edges, where nodes represent genes and edges represent their interactions. Topological analysis of the PPI network was conducted using the MCC algorithm.

### GSEA analysis

**Fig. 5:**
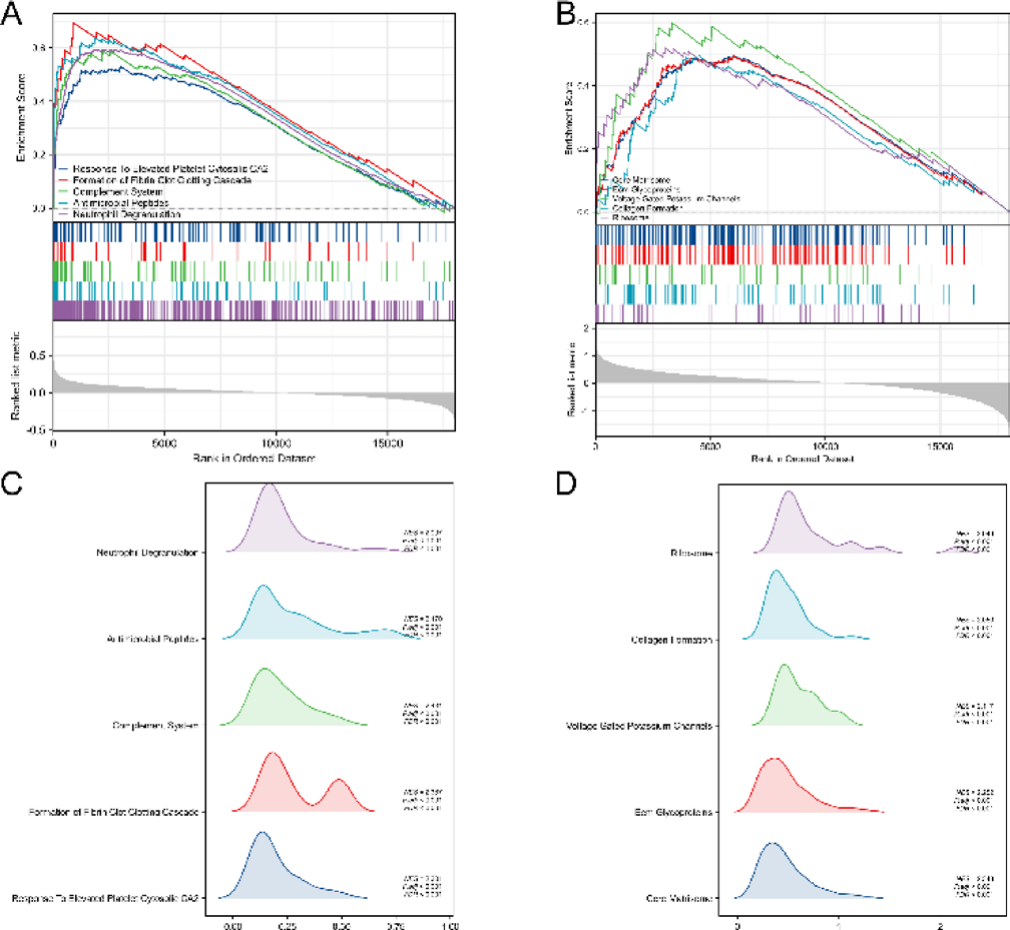
Representative GSEA enrichment plots of DEGs between disease group(depression and renal failure) and normal group expression groups. (**A**) The first 5 significantly enriched GSEA in depression. (**B**) The first 5 significantly enriched GSEA in renal failure. (**C**,**D**) Visualizing the results of GSEA enrichment analysis in the depression and failure group using ridge plots.

We assessed the pertinent signaling pathways using GSEA analysis based on the differential expression of all genes in the illness group compared to the normal group. The top 5 signaling pathways in the depression and renal failure groups are shown in Fig. 5A-B. The ridge plots shown in Fig. 5C-D then represent the distribution of significant pathways in depression and renal failure groups by the height of the peaks, with the more densely distributed intervals having higher peaks.

The results showed that in the depression group, Neutrophil Degranulation, Antimicrobial Peptides, Complement System, Formation of Fibrin Clot Clotting Cascade, Response To Elevated Platelet Cytosolic CA2, and other immune-related pathways were significantly enriched. In contrast, the renal failure group significantly enriched the ribosome Collagen Formation, Voltage-Gated Potassium Channels, Ecm Glycoproteins, Core Matrisome, and other important physiological pathways. These GSEA pathway analyses suggest that immune-related biological pathways likely influence the disease characteristics of the depression and renal failure groups.

### Immune cell infiltration analysis

**Fig. 6:**
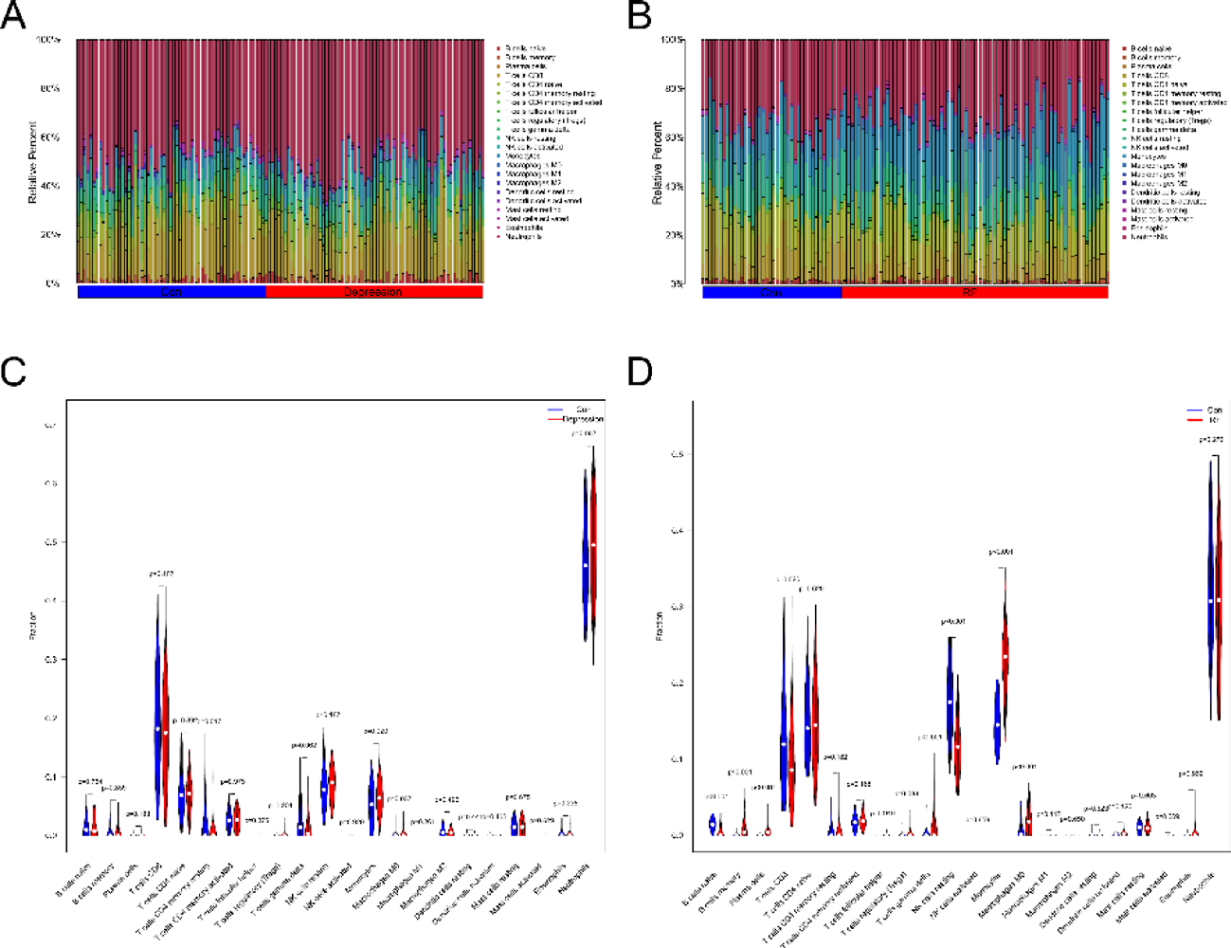
CIBERSORT analysis of immune infiltration. The fraction of 22 subsets of immune cells in the depression group and renal failure group. Depression(**A**), renal failure(**B**). X-axis: each GEO sample; Y-axis: percentage of each kind of immune cell. The violin plot shows the immune infiltration in depression(C) and renal failure(D), where blue represents the control group and red represents the disease group.

Figure 6A-B shows the heat map of immune cell infiltration of individual samples in all GSE98793 and GSE37171 datasets included in the study. Compared with the normal group, the depression group has higher gamma delta T cell infiltration, lower monocytes, and M0 macrophage infiltration (Fig. 6C). In contrast, the renal failure group had higher immune infiltration on naive B cells, CD8 + T cells, and Resting NK cells and lower immune infiltration on B memory cells, Plasma, T regulatory cells (Tregs), gamma delta T cells, and M0 macrophages (Fig. 6D).

### Data validation

**Fig. 7:**
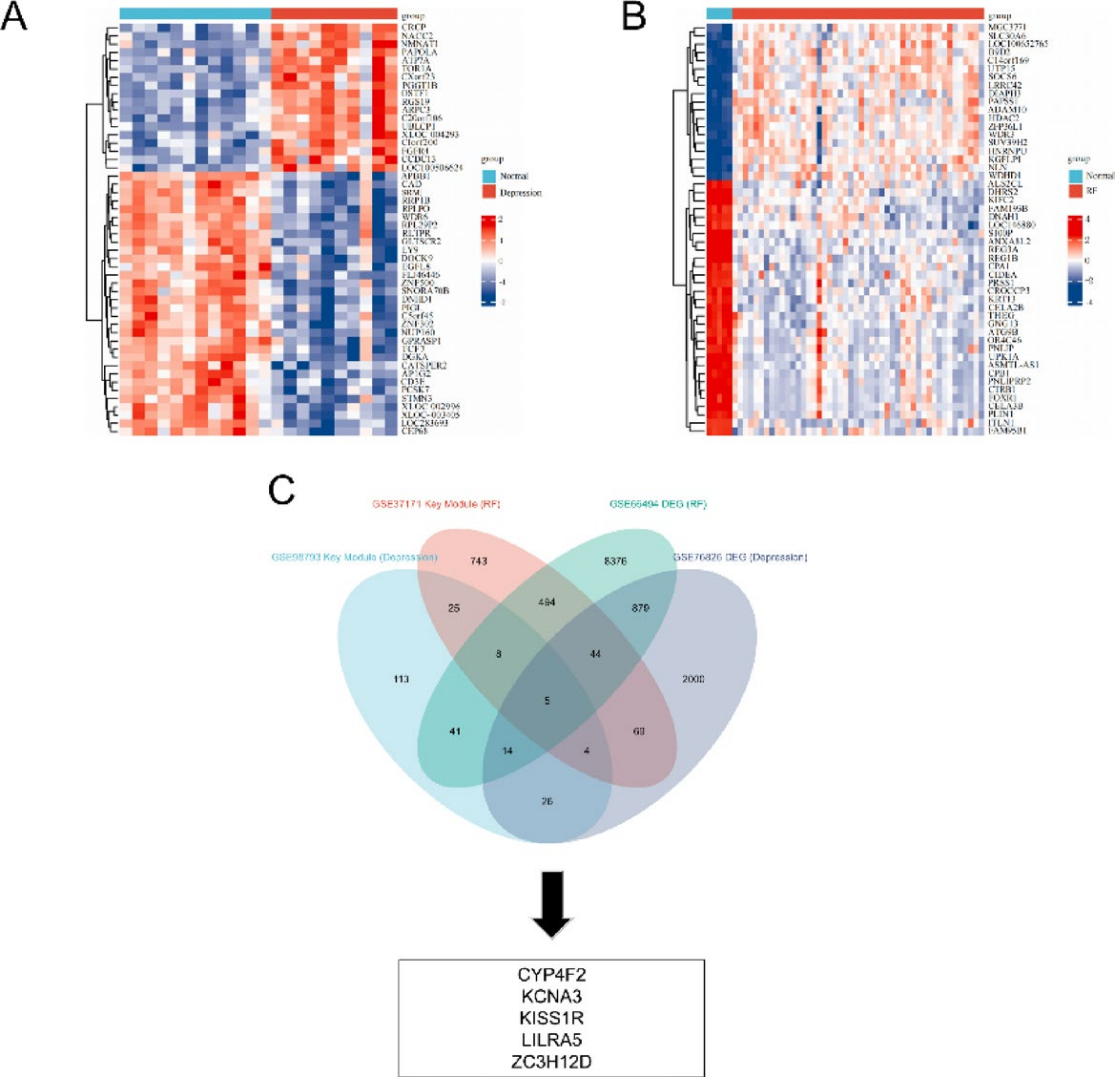
Validation of hub genes. (**A**) A heat map of hierarchical clustering of DEGs in the GSE76826 dataset. (**B**) A heat map of hierarchical clustering of DEGs in the GSE66494 dataset. (**C**) Identification of overlapping genes between differentially expressed genes in GSE76826 and GSE66494.

Finally, additional datasets related to depression (GSE76826) and renal failure (GSE66494) from the GEO database were used to validate the identified hub genes. Figure 7A and B present hierarchical clustering heatmaps of differentially expressed genes (DEGs) in the respective datasets. Differential expression was determined using a threshold of |log₂ fold change| > 0.4 and nominal *P* < 0.05. This relatively moderate log₂ fold change threshold has been described in previous studies^24^ and was chosen to ensure that genes with smaller expression changes were not overlooked. While adjusted p-values are commonly used, the application of nominal p-values is also prevalent in exploratory analyses to identify more potential candidates for further validation.

**Fig. 8:**
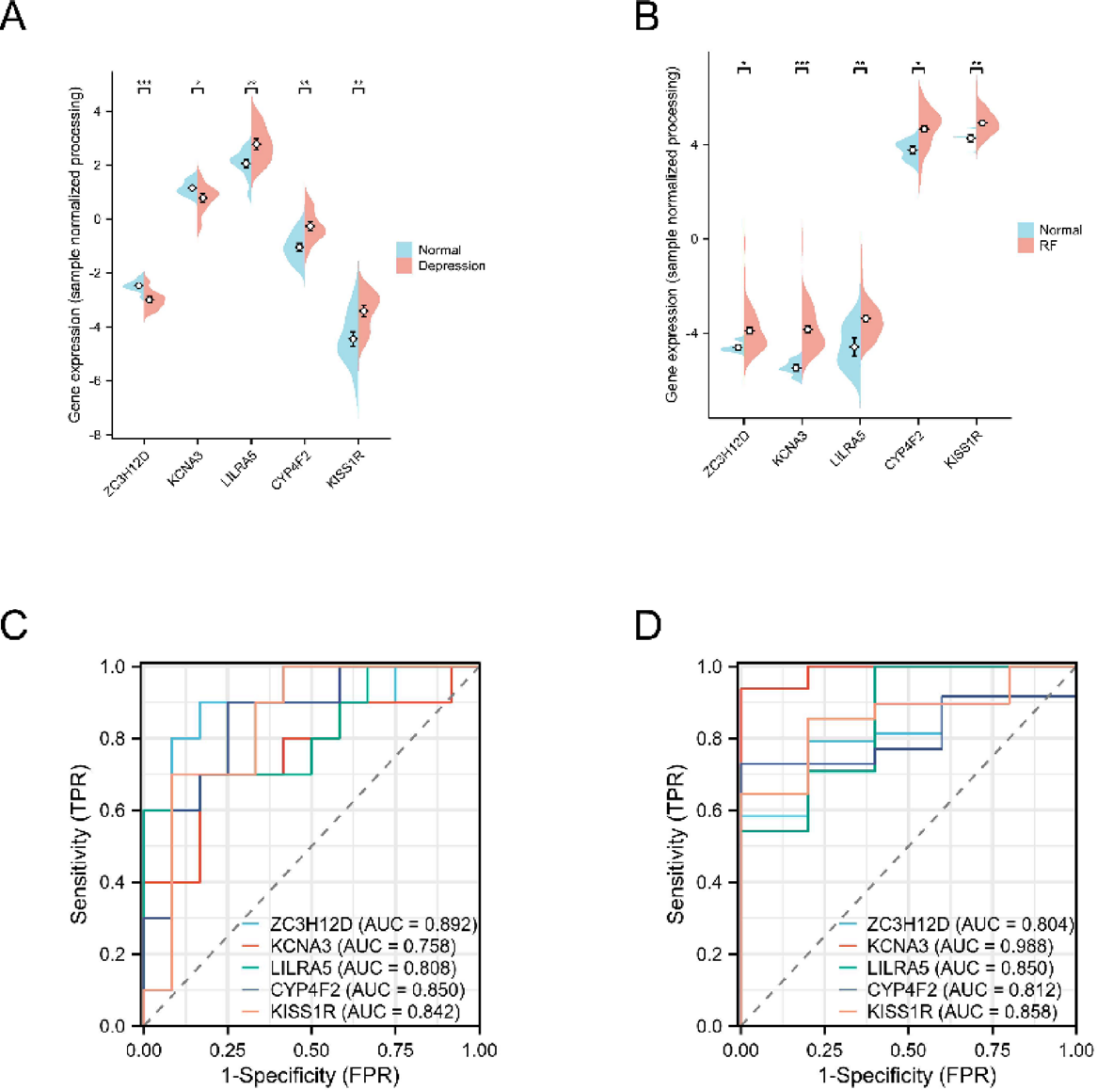
Differential expression profiles of hub genes with potential diagnostic ROC curves. (**A**) Differential expression of CYP4F2, KCNA3, KISS1R, LILRA5, and ZC3H12D between the depressed and normal groups (GSE76826) (**B**) Differential expression of CYP4F2, KCNA3, KISS1R, LILRA5, and ZC3H12D between the renal failure and normal groups (GSE66494). (**C**,**D**) ROC curve showing the diagnostic performance of the signature genes. RF, renal failure.

We discovered that the five key genes CYP4F2, KCNA3, KISS1R, LILRA5 and ZC3H12D remained different after Venn diagram overlap of DEGs across depression and renal failure datasets (Fig. 7C). The GeneCards database displays more information regarding the selected hub genes (Table 2). Expression of the screened signature genes significantly differed between the depression and chronic nephritis groups, indicating that these genes may have a role in depression and chronic nephritis (Fig. 8A-B). The receiver operating characteristic (ROC) analysis demonstrated that the signature genes displayed varying diagnostic performance across both the depression and renal failure groups (Fig. 8C-D). In both groups, the genes showed substantial potential for distinguishing between disease and control conditions, with notable differences in performance across the genes. Notably, LILRA5, CYP4F2, and KISS1R consistently exhibited higher expression levels in the disease groups compared to controls, suggesting their prominent roles in the pathophysiology of depression and chronic nephritis. Moreover, the expression profiles of these five hub genes and ROC analysis in the initial datasets (GSE98793 and GSE37171) were shown in Supplementary Fig. 2A-D. The expression patterns of these genes were generally consistent with those observed in the validation datasets.

**Table 2:** The hub genes in depression and renal failure.

Gene	GeneCards Identifier	Full Name	Gene-related Diseases
CYP4F2	GC19M016474	Cytochrome P450 Family 4 Subfamily F Member 2	Atrial Fibrillation, Thrombosis, Coumarin Resistance
KCNA3	GC01M111630	Potassium Voltage-Gated Channel Subfamily A Member 3	Eye Degenerative Disease, Sensory System Disease, Developmental And Epileptic Encephalopathy
KISS1R	GC19P000917	KISS1 Receptor	Hypogonadism, Uterine Inflammatory Disease, Leydig Cell Hypoplasia
LILRA5	GC19M054307	Leukocyte Immunoglobulin Like Receptor A5	-
ZC3H12D	GC06M149446	Zinc Finger CCCH-Type Containing 12D	Follicular Lymphoma, Patellofemoral Pain Syndrome, Large B-Cell Lymphoma

### Identification of drugs associated with hub genes

The drug-gene interaction analysis focused on LILRA5, CYP4F2, and KISS1R, which are implicated in the comorbidity of depression and renal failure. As detailed in Table 3, several drugs previously associated with these genes were identified through the DSigDB library in Enrichr. These results highlight potential pharmacological agents that may modulate the activity of these genes. The interactions listed in Table 3 were evaluated based on their statistical significance, effect sizes, and combined scores, offering a reference point for future experimental validation.

**Table 3:** Prediction of hub genes-Related Drugs.

Term	*P*-value	Adjusted *P*-value	Odds Ratio	Combined Score	Target
R-atenolol PC3 UP	2.85E-04	0.016518474	204.1546392	1666.661089	CYP4F2;LILRA5
Phylloquinone CTD 00006555	0.002098597	0.043403841	768.6153846	4739.656184	CYP4F2
IN1541 CTD 00001481	0.003446153	0.043403841	453.9772727	2574.276639	CYP4F2
inositol 1-phosphate BOSS	0.003595807	0.043403841	434.2173913	2443.769793	KISS1R
Dynorphin A porcine BOSS	0.004194272	0.043403841	369.8148148	2024.379431	KISS1R
warfarin CTD 00005716	0.00524101	0.043403841	293.5735294	1541.625367	CYP4F2
zalcitabine PC3 UP	0.005912607	0.043403841	42.43777778	217.7341658	CYP4F2;LILRA5
benzethonium chloride CTD 00005485	0.006137631	0.043403841	249.4625	1270.591461	KISS1R
dorsomorphin CTD 00004649	0.006735079	0.043403841	226.7386364	1133.789719	CYP4F2
ketoconazole CTD 00006190	0.011059423	0.064144652	136.4657534	614.7062292	CYP4F2

## Discussion

Utilizing the large prospective cohort from the UK Biobank and the gene expression data from GEO, the present study revealed a bidirectional association that depression is associated with an increased risk of renal failure and renal failure is associated with an increased risk of depression. The related pathways include acute inflammatory response, specific granule lumen, and immune receptor activity. PPI network analysis identified CYP4F2, KCNA3, KISS1R, LILRA5, and ZC3H12D as key players in the shared pathophysiology of ESRD and depression. Validation studies further emphasized the roles of LILRA5, CYP4F2, and KISS1R in these mechanisms. Briefly, depression and renal failure are not simply concurrent conditions but instead appear to influence each other in a bidirectional manner.

Our main results was consistent with previous studies that depressive symptoms increase the risk of CKD by 38% in middle-aged and older adults, with a dose-dependent rise in depression risk as eGFR declines (*P* < 0.01)^31,32^. Despite the clinical relevance of this comorbidity, the molecular mechanisms underlying the relationship between depression and renal failure remain poorly understood. Using WGCNA, we identified key modules shared between depression and renal failure. Functional enrichment analysis revealed significant biological processes, including the acute inflammatory response, defense responses to bacterial components, and immune receptor activity, which may contribute to the comorbidity of these conditions. Further, protein-protein interaction (PPI) network analysis pinpointed five central genes CYP4F2, KCNA3, KISS1R, LILRA5, and ZC3H12D as pivotal players in the shared pathophysiology of depression and renal failure.

The bidirectional relationship of depression with CKD may be explained by several mechanisms. Immune-related processes, including inflammatory responses and immune receptor activity, are likely among the most important. A chronic inflammatory state is common in CKD patients, with persistent release of pro-inflammatory cytokines such as IL-6 and TNF-α^17,20^. These cytokines can activate microglia in the brain, triggering neuroinflammation and leading to depressive symptoms. On the other hand, depression is associated with elevated peripheral inflammatory markers such as C-reactive protein (CRP), which may exacerbate local renal inflammation and contribute to kidney damage^33,34^. Arachidonic acid metabolism plays a crucial role in both kidney disease and depression. In CKD, aberrant arachidonic acid metabolism via the cytochrome P450 (CYP450) and cyclooxygenase (COX) pathways leads to the production of pro-inflammatory mediators such as leukotrienes and prostaglandin E2, which promote glomerulosclerosis and interstitial fibrosis; the pathway is also implicated in the central nervous system, where prostaglandin E2 crosses the blood-brain barrier and inhibits serotonin synthesis, potentially contributing to depressive behaviour^35–37^. Animal models have shown that the inhibition of arachidonic acid metabolism reduces both renal inflammation and depressive-like symptoms, supporting the role of this pathway in the comorbidity of these diseases^38^. However, the causality or directionality of the mechanisms driving the comorbidity remains unclear and may need further investigation.

Among the differentially expressed genes identified, LILRA5 was predominantly expressed in macrophages and associated with the common γ-chain of Fc receptors (FcR)^39,40^. Crosslinking of LILRA5 induces the phosphorylation of Src tyrosine kinase and spleen tyrosine kinase, thereby influencing macrophage activation states^41^. These findings suggest that LILRA5-mediated signaling pathways may play a role in the inflammatory processes underlying both depression and renal failure. Our immune infiltration analysis further revealed an increase in M0 macrophages, which may indicate a shift in macrophage polarization, potentially contributing to the inflammatory microenvironment in these conditions. In addition, KISS1R (Kisspeptin-1 receptor, also known as GPR54), a G protein-coupled receptor, was significantly upregulated. Previous studies have shown that KISS1R modulates the hypothalamic-pituitary-gonadal axis by regulating GnRH neurons in the hypothalamus. Overactivation of KISS1R can disrupt GnRH secretion, potentially exacerbating depressive symptoms through its effects on neurotransmitter regulation^42,43^. Furthermore, CYP4F2, a cytochrome P450 enzyme primarily expressed in the human liver, was highlighted in our analysis. Recent research indicates that metabolites mediated by CYP4F2, particularly 13’-COOH and γ-CEHC derived from γ-tocopherol, exhibit stronger anti-inflammatory activity compared to α-tocopherol (αT). This suggests that CYP4F2 may play a protective role by mitigating inflammation in these conditions^44,45^. In addition, we examined the expression profiles of these five hub genes in the initial datasets. The expression trends of these genes showed general consistency, except for KCNA3 in depression and ZC3H12D and CYP4F2 in renal failure exhibiting opposite expression patterns. The differential expression and AUC values in these datasets were generally lower, suggesting that cohort differences, sample heterogeneity, and technical variations may contribute to these discrepancies. These findings emphasize the need for further validation in independent cohorts.

Another shared pathway involves serotonin mechanisms, which are linked to major depressive disorder (MDD) and cardiovascular risk. Increased platelet size and number in MDD patients may contribute to a thrombotic phenotype, contrasting with the platelet dysfunction commonly observed in ESRD patients undergoing hemodialysis^46,47^. This paradox could partially explain the distinct bleeding tendencies in ESRD patients, which may also involve impaired platelet-dependent thrombosis. GSEA analysis revealed elevated immune responses, particularly neutrophil degranulation, in depressed patients. Studies have demonstrated increased neutrophil counts and a pronounced inflammatory response during the active phase of depression, evidenced by changes in white blood cell differentials^48^.

Similarly, neutrophil dysfunction is prevalent in ESRD. Although neutrophil counts are elevated, their capacity for superoxide production, phagocytosis, chemotaxis, and degranulation is impaired in uremic patients^49^. Moreover, excessive or untimely release of apoptotic reactive oxygen species (ROS), neutrophil extracellular traps (NETs), and granule proteins can cause cytotoxic effects, tissue damage, and chronic inflammation, further exacerbating disease pathology^50^.

Taking advantage of the UKB study, this study systematically evaluated the association of depression with the renal failure and possessed several strengths, including its longitudinal design and large sample size, which ensured a sufficient number of cases for reliable data analysis. However, some limitations should be considered when interpreting these findings. Firstly, the UK Biobank cohort mainly comprises participants of European descent, limiting our findings’ generalizability to other ethnic groups. Secondly, this study focused primarily on modules or DEGs positively correlated with disease conditions as these modules typically represented the upregulation in response to inflammation or immune activation. However, the potential biological significance of negatively correlated modules may be overlooked. Since negatively correlated modules may also potentially reflect suppressed protective functions in the disease state, future studies should explore these modules to fully understand their possible roles. Thirdly, although this study has revealed the bidirectional relationship between depression and CKD, the causality and directionality underlying this comorbidity remain unclear. While we cannot establish causal relationships in this study, future studies like Mendelian randomization approaches may offer a more robust framework to better address causality between these conditions.

## Conclusions

In conclusion, ribosome stability, oxidative phosphorylation, neutrophil activity, and macrophage dynamics are key factors linking renal failure and depression. Our analysis identified 23 common genes involved in the shared pathophysiology, with LILRA5, CYP4F2, and KISS1R emerging as potential therapeutic and diagnostic targets. These findings lay the groundwork for future research into the molecular mechanisms of this comorbidity. Furthermore, immunotherapeutic strategies, such as monocyte activation and reinfusion, may offer novel treatment approaches to improve outcomes in patients with both conditions.

## Electronic supplementary material

Below is the link to the electronic supplementary material.

Supplementary Material 1

Supplementary Material 2

Supplementary Material 3

Supplementary Material 4

## Data Availability

The data sets used and analyzed during the current study are available from the public Gene Expression Omnibus (GEO) database. The data sets supporting the findings of this study are openly available. The analytical research data referenced in this study are contained within the following GEO data sets: GSE98793 and GSE37171. The validation data utilized are contained within GEO data sets: GSE76826 and GSE66494. These data sets can be accessed through the GEO database portal at https://www.ncbi.nlm.nih.gov/geo/.
