# Supplementary material for: Integrated analysis identifies key genes underlying the bidirectional association between depression and renal failure
Source: Sci Rep. 2025 Jul 1;15:21279. doi: 10.1038/s41598-025-04707-9 (PMC12219552; doi:10.1038/s41598-025-04707-9)
Supplement: Supplementary file 1 — Supplementary Material 1 [file 41598_2025_4707_MOESM1_ESM.docx]

#if (!requireNamespace("BiocManager", quietly = TRUE))

# install.packages("BiocManager")

#BiocManager::install(c("GO.db", "preprocessCore", "impute","limma"))

#install.packages(c("matrixStats", "Hmisc", "foreach", "doParallel", "fastcluster", "dynamicTreeCut", "survival"))

#install.packages("WGCNA")

library("WGCNA") # Load WGCNA package

library("limma") # Load limma package

expFile="geneMatrix.txt" # Input file

conFile="sample1.txt" # Control group samples

treatFile="sample2.txt" # Treatment group samples

# Read input files and process them

rt=read.table(expFile,sep="\t",header=T,check.names=F)

rt=as.matrix(rt)

rownames(rt)=rt[,1]

exp=rt[,2:ncol(rt)]

dimnames=list(rownames(exp),colnames(exp))

data=matrix(as.numeric(as.matrix(exp)),nrow=nrow(exp),dimnames=dimnames)

data=avereps(data)

#data=log2(data+1) # If the input data values are large, take the log2 of the data by uncommenting this line

data=normalizeBetweenArrays(data)

data=data[apply(data,1,sd)>0.5,] # Remove genes with low variance

# Read sample information

sample1=read.table(conFile,sep="\t",header=F,check.names=F)

sample2=read.table(treatFile,sep="\t",header=F,check.names=F)

conData=data[,as.vector(sample1[,1])]

treatData=data[,as.vector(sample2[,1])]

data=cbind(conData,treatData)

normalCount=ncol(conData)

tumorCount=ncol(treatData)

datExpr0=t(data)

### Check for missing values

gsg = goodSamplesGenes(datExpr0, verbose = 3)

if (!gsg$allOK)

{

# Optionally, print the gene and sample names that were removed:

if (sum(!gsg$goodGenes)>0)

printFlush(paste("Removing genes:", paste(names(datExpr0)[!gsg$goodGenes], collapse = ", ")))

if (sum(!gsg$goodSamples)>0)

printFlush(paste("Removing samples:", paste(rownames(datExpr0)[!gsg$goodSamples], collapse = ", ")))

# Remove the offending genes and samples from the data:

datExpr0 = datExpr0[gsg$goodSamples, gsg$goodGenes]

}

### Sample clustering

sampleTree = hclust(dist(datExpr0), method = "average")

pdf(file = "1_sample_cluster.pdf", width = 12, height = 9)

par(cex = 0.6)

par(mar = c(0,4,2,0))

plot(sampleTree, main = "Sample clustering to detect outliers", sub="", xlab="", cex.lab = 1.5, cex.axis = 1.5, cex.main = 2)

### Add cut line

abline(h = 40, col = "red")

dev.off()

### Remove samples below the cut line

clust = cutreeStatic(sampleTree, cutHeight = 50, minSize = 10)

table(clust)

keepSamples = (clust==1)

datExpr0 = datExpr0[keepSamples, ]

### Prepare clinical data

traitData=data.frame(Normal=c(rep(1,normalCount),rep(0,tumorCount)),

Tumor=c(rep(0,normalCount),rep(1,tumorCount)))

row.names(traitData)=colnames(data)

fpkmSamples = rownames(datExpr0)

traitSamples =rownames(traitData)

sameSample=intersect(fpkmSamples,traitSamples)

datExpr0=datExpr0[sameSample,]

datTraits=traitData[sameSample,]

### Sample clustering

sampleTree2 = hclust(dist(datExpr0), method = "average")

traitColors = numbers2colors(datTraits, signed = FALSE)

pdf(file="2_sample_heatmap.pdf",width=12,height=12)

plotDendroAndColors(sampleTree2, traitColors,

groupLabels = names(datTraits),

main = "Sample dendrogram and trait heatmap")

dev.off()

### Power value scatter plot

enableWGCNAThreads() # Enable multi-threading

powers = c(1:20) # Range of power values 1:20

sft = pickSoftThreshold(datExpr0, powerVector = powers, verbose = 5)

pdf(file="3_scale_independence.pdf",width=9,height=5)

par(mfrow = c(1,2))

cex1 = 0.9

### Scatter plot of fit index vs. power

plot(sft$fitIndices[,1], -sign(sft$fitIndices[,3])*sft$fitIndices[,2],

xlab="Soft Threshold (power)",ylab="Scale Free Topology Model Fit,signed R^2",type="n",

main = paste("Scale independence"));

text(sft$fitIndices[,1], -sign(sft$fitIndices[,3])*sft$fitIndices[,2],

labels=powers,cex=cex1,col="red");

abline(h=0.90,col="red") # Can modify this threshold

### Scatter plot of mean connectivity vs. power

plot(sft$fitIndices[,1], sft$fitIndices[,5],

xlab="Soft Threshold (power)",ylab="Mean Connectivity", type="n",

main = paste("Mean connectivity"))

text(sft$fitIndices[,1], sft$fitIndices[,5], labels=powers, cex=cex1,col="red")

dev.off()

### Adjacency matrix conversion

sft # View best power value

softPower =sft$powerEstimate # Best power value (can be specified as a lower value for co-morbidity analysis for comparison)

adjacency = adjacency(datExpr0, power = softPower)

softPower

### TOM matrix

TOM = TOMsimilarity(adjacency)

dissTOM = 1-TOM

### Gene clustering

geneTree = hclust(as.dist(dissTOM), method = "average");

pdf(file="4_gene_clustering.pdf",width=12,height=9)

plot(geneTree, xlab="", sub="", main = "Gene clustering on TOM-based dissimilarity",

labels = FALSE, hang = 0.04)

dev.off()

### Dynamic cut module detection

minModuleSize = 30 # Minimum number of genes in a module

dynamicMods = cutreeDynamic(dendro = geneTree, distM = dissTOM,

deepSplit = 2, pamRespectsDendro = FALSE,

minClusterSize = minModuleSize);

table(dynamicMods)

dynamicColors = labels2colors(dynamicMods)

table(dynamicColors)

pdf(file="5_Dynamic_Tree.pdf",width=8,height=6)

plotDendroAndColors(geneTree, dynamicColors, "Dynamic Tree Cut",

dendroLabels = FALSE, hang = 0.03,

addGuide = TRUE, guideHang = 0.05,

main = "Gene dendrogram and module colors")

dev.off()

### Similar module clustering

MEList = moduleEigengenes(datExpr0, colors = dynamicColors)

MEs = MEList$eigengenes

MEDiss = 1-cor(MEs);

METree = hclust(as.dist(MEDiss), method = "average")

pdf(file="6_Clustering_module.pdf",width=7,height=6)

plot(METree, main = "Clustering of module eigengenes",

xlab = "", sub = "")

MEDissThres = 0.25 # Cut height can be adjusted

abline(h=MEDissThres, col = "red")

dev.off()

### Merge similar modules

merge = mergeCloseModules(datExpr0, dynamicColors, cutHeight = MEDissThres, verbose = 3)

mergedColors = merge$colors

mergedMEs = merge$newMEs

pdf(file="7_merged_dynamic.pdf", width = 9, height = 6)

plotDendroAndColors(geneTree, mergedColors,"Dynamic Tree Cut",

dendroLabels = FALSE, hang = 0.03,

addGuide = TRUE, guideHang = 0.05,

main = "Gene dendrogram and module colors(GEO)")

dev.off()

moduleColors = mergedColors

table(moduleColors)

colorOrder = c("grey", standardColors(50))

moduleLabels = match(moduleColors, colorOrder)-1

MEs = mergedMEs

### Module-trait heatmap

nGenes = ncol(datExpr0)

nSamples = nrow(datExpr0)

moduleTraitCor = cor(MEs, datTraits, use = "p")

moduleTraitPvalue = corPvalueStudent(moduleTraitCor, nSamples)

pdf(file="8_Module_trait.pdf",width=6,height=6)

textMatrix = paste(signif(moduleTraitCor, 2), "\n(",

signif(moduleTraitPvalue, 1), ")", sep = "")

dim(textMatrix) = dim(moduleTraitCor)

par(mar =
