# Supplementary material for: Integrated analysis identifies key genes underlying the bidirectional association between depression and renal failure
Source: Sci Rep. 2025 Jul 1;15:21279. doi: 10.1038/s41598-025-04707-9 (PMC12219552; doi:10.1038/s41598-025-04707-9)
Supplement: Supplementary file 4 — Supplementary Material 4 [file 41598_2025_4707_MOESM4_ESM.docx]

**Supplementary Table 1. Definitions of Variables for Data Extraction from the UK Biobank**

| **Variables** | **Definition** |
| --- | --- |
|  |  |
| Incident renal failure | ICD-10 code: N17-N19, E85.3, N16.5, Q60.1, T82.4, T86.1, Y60.2, Y61.2, Y62.2, Y84.1, Z49.0, Z49.1, Z49.2, Z94.0, and Z99.2 |
| Prevalent renal failure | ICD-10 code: N17-N19, E85.3, N16.5, Q60.1, T82.4, T86.1, Y60.2, Y61.2, Y62.2, Y84.1, Z49.0, Z49.1, Z49.2, Z94.0, and Z99.2 |
| Incident depression | ICD-10: F31.3, F31.4, F31.5, F32, F33 |
| Prevalent depression | ICD-10: F31.3, F31.4, F31.5, F32, F33  Depression scores >=3 (measured using the Patient Health Questionnaire-2) |

**Supplementary Table 2. Genes in Key Modules Associated with Depression and Renal Failure**

| **Genes** |
| --- |
|  |
| GZMB, LAX1, LILRA5, LINC01000, LINC01001, LINC01002, LOC100132062, LOC100133182, LOC100133331, LOC101929819, LOC729737, RP11-44F14.8, S100A12, SLPI, UQCRH, UQCRHL, ALPL, AV8S2, TRAV13-2, CAMP, CCR7, CD177, CYSTM1, FFAR2, HP, HPR, KCNA3, KISS1R, KLRD1, LIAS, LINC00969, LINC01347, LOC101060494, LOC101926894, LOC101929038, LOC101930127, LOC101930567, LOC388572, LINC01127, LOC100506076, LOC100506123, LOC101927268, LOC102724851, ORM1, ORM2, PGLYRP1, POLE3, RP11-28F1.2, T-CellReceptorV-alpharegion, TRAV9-2, TCN1, TCRAVN1, TRAV25, TCRDV2, TRDC, YME1L1, ZC3H12D, CYP4F2, CYP4F3, EPHB4, MPO, RPH3A, TCR-alpha, TRAV12-3, TRDV3 |

**Supplementary Table 3. Key Genes and Pathways in Depression-Renal Failure Comorbidity (GO/KEGG Analysis)**

| ONTOLOGY | ID | Description | Gene  Ratio | Bg  Ratio | pvalue | p.adjust | qvalue | geneID | Count |
| --- | --- | --- | --- | --- | --- | --- | --- | --- | --- |
| BP | GO:0002526 | acute inflammatory response | 6/37 | 113/18800 | 8.23835E-08 | 6.37648E-05 | 4.96035E-05 | CCR7/FFAR2/HP/HPR/ORM1/ORM2 | 6 |
| BP | GO:0042742 | defense response to bacterium | 7/37 | 364/18800 | 6.01005E-06 | 0.002325889 | 0.001809341 | S100A12/SLPI/CAMP/HP/PGLYRP1/TRDC/MPO | 7 |
| BP | GO:0006959 | humoral immune response | 6/37 | 317/18800 | 3.28021E-05 | 0.006981748 | 0.005431198 | S100A12/SLPI/CAMP/CCR7/PGLYRP1/TRDC | 6 |
| BP | GO:0032496 | response to lipopolysaccharide | 6/37 | 333/18800 | 4.31849E-05 | 0.006981748 | 0.005431198 | SLPI/ALPL/CAMP/CCR7/LIAS/MPO | 6 |
| BP | GO:0002237 | response to molecule of bacterial origin | 6/37 | 354/18800 | 6.06582E-05 | 0.006981748 | 0.005431198 | SLPI/ALPL/CAMP/CCR7/LIAS/MPO | 6 |
| CC | GO:0035580 | specific granule lumen | 7/37 | 62/19594 | 2.13999E-11 | 2.11859E-09 | 1.35157E-09 | SLPI/CAMP/HP/ORM1/ORM2/PGLYRP1/TCN1 | 7 |
| CC | GO:0042581 | specific granule | 8/37 | 160/19594 | 5.23509E-10 | 2.59137E-08 | 1.65319E-08 | SLPI/CAMP/CD177/HP/ORM1/ORM2/PGLYRP1/TCN1 | 8 |
| CC | GO:0034774 | secretory granule lumen | 9/37 | 322/19594 | 6.491E-09 | 1.46938E-07 | 9.37404E-08 | S100A12/SLPI/CAMP/HP/ORM1/ORM2/PGLYRP1/TCN1/MPO | 9 |
| CC | GO:0060205 | cytoplasmic vesicle lumen | 9/37 | 325/19594 | 7.03591E-09 | 1.46938E-07 | 9.37404E-08 | S100A12/SLPI/CAMP/HP/ORM1/ORM2/PGLYRP1/TCN1/MPO | 9 |
| CC | GO:0031983 | vesicle lumen | 9/37 | 327/19594 | 7.42111E-09 | 1.46938E-07 | 9.37404E-08 | S100A12/SLPI/CAMP/HP/ORM1/ORM2/PGLYRP1/TCN1/MPO | 9 |
| MF | GO:0046906 | tetrapyrrole binding | 4/31 | 149/18410 | 0.000109384 | 0.013235447 | 0.009441554 | TCN1/CYP4F2/CYP4F3/MPO | 4 |
| MF | GO:0008391 | arachidonic acid monooxygenase activity | 2/31 | 21/18410 | 0.000564878 | 0.032411449 | 0.023120825 | CYP4F2/CYP4F3 | 2 |
| MF | GO:0070330 | aromatase activity | 2/31 | 25/18410 | 0.00080359 | 0.032411449 | 0.023120825 | CYP4F2/CYP4F3 | 2 |
| MF | GO:0020037 | heme binding | 3/31 | 139/18410 | 0.001621758 | 0.041477742 | 0.029588298 | CYP4F2/CYP4F3/MPO | 3 |
| MF | GO:0140375 | immune receptor activity | 3/31 | 148/18410 | 0.001940276 | 0.041477742 | 0.029588298 | LILRA5/CCR7/KLRD1 | 3 |
| KEGG | hsa05332 | Graft-versus-host disease | 2/17 | 42/8164 | 0.003346174 | 0.003346174 | 0.130324665 | GZMB/KLRD1 | 2 |
| KEGG | hsa00590 | Arachidonic acid metabolism | 2/17 | 61/8164 | 0.006948835 | 0.006948835 | 0.135319426 | CYP4F2/CYP4F3 | 2 |
| KEGG | hsa04260 | Cardiac muscle contraction | 2/17 | 87/8164 | 0.013761626 | 0.013761626 | 0.176175096 | UQCRH/UQCRHL | 2 |
| KEGG | hsa04650 | Natural killer cell mediated cytotoxicity | 2/17 | 131/8164 | 0.029689681 | 0.029689681 | 0.176175096 | GZMB/KLRD1 | 2 |
| KEGG | hsa00730 | Thiamine metabolism | 1/17 | 15/8164 | 0.03080953 | 0.03080953 | 0.176175096 | ALPL | 1 |


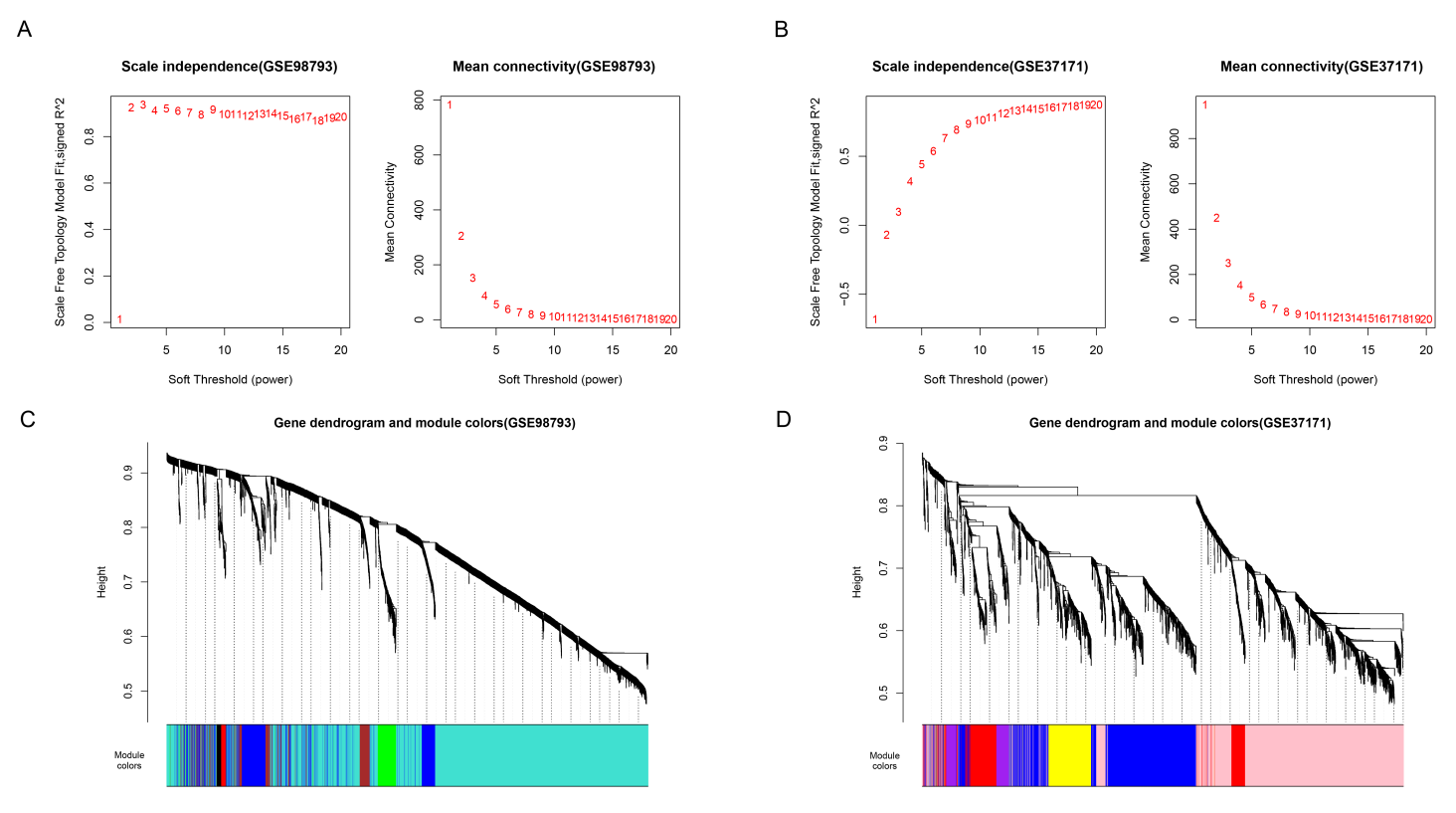


**Supplementary Figure 1: The co-expression modules analysis.** (A-B) In the left panel, the x-axis shows the soft-threshold power, and the y-axis reflects the scale-free topology model fit index. In the right panel, the x-axis shows the soft-threshold power, and the y-axis reflects the mean connectivity (degree). (C-D) Clustering dendrogram of genes with assigned module colors. Dendrogram of all differentially expressed genes clustered based on the dissimilarity measurement (1-TOM). According to the Dynamic Tree Cut, the colored row underneath the dendrogram indicates the module assignment.


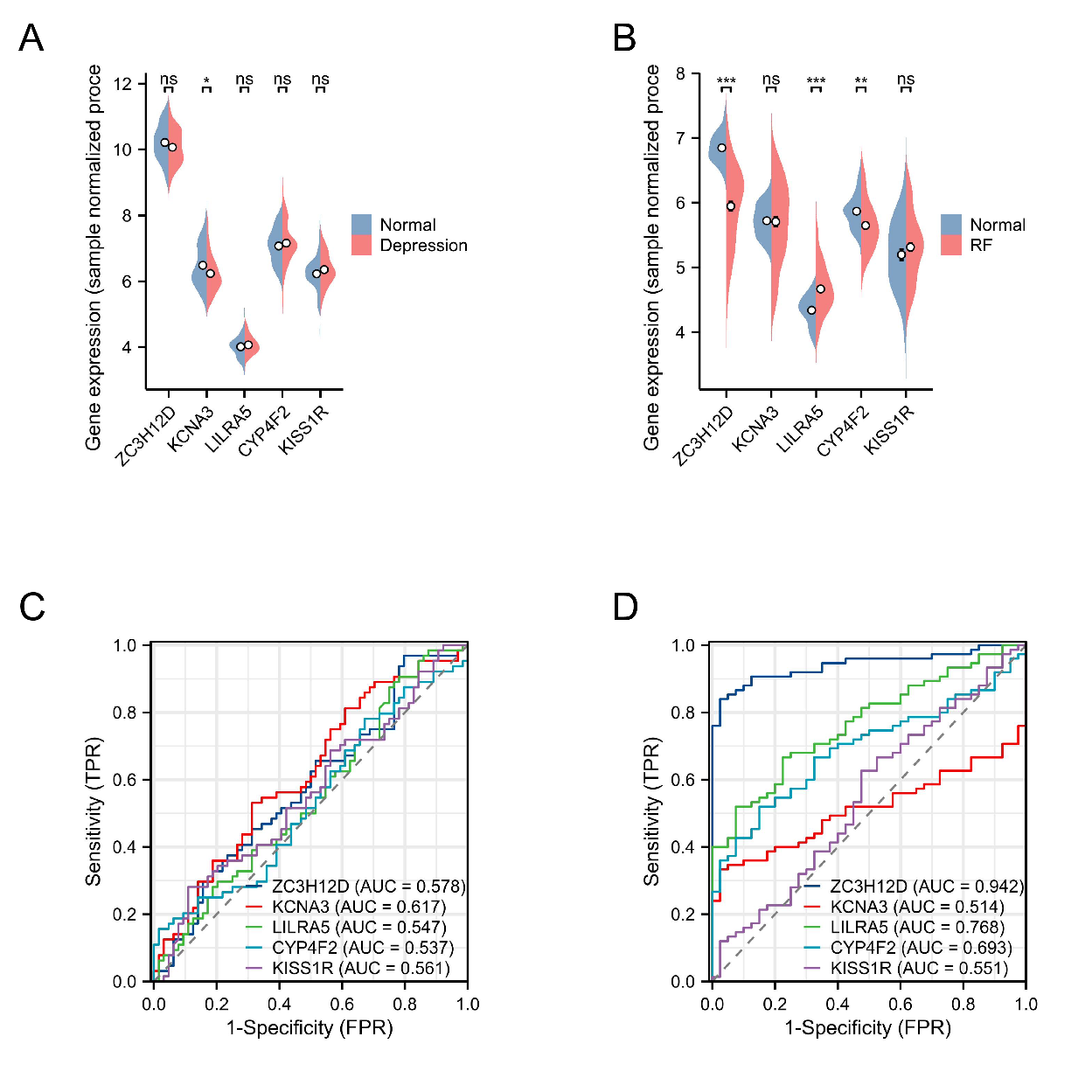


**Supplementary Figure 2: Differential expression profiles of hub genes with potential diagnostic ROC curves**. (A) Differential expression of CYP4F2, KCNA3, KISS1R, LILRA5, and ZC3H12D between the depressed and normal groups (GSE98793) (B) Differential expression of CYP4F2, KCNA3, KISS1R, LILRA5, and ZC3H12D between the renal failure and normal groups (GSE37171). (C-D) ROC curve showing the diagnostic performance of the signature genes. RF, renal failure.
